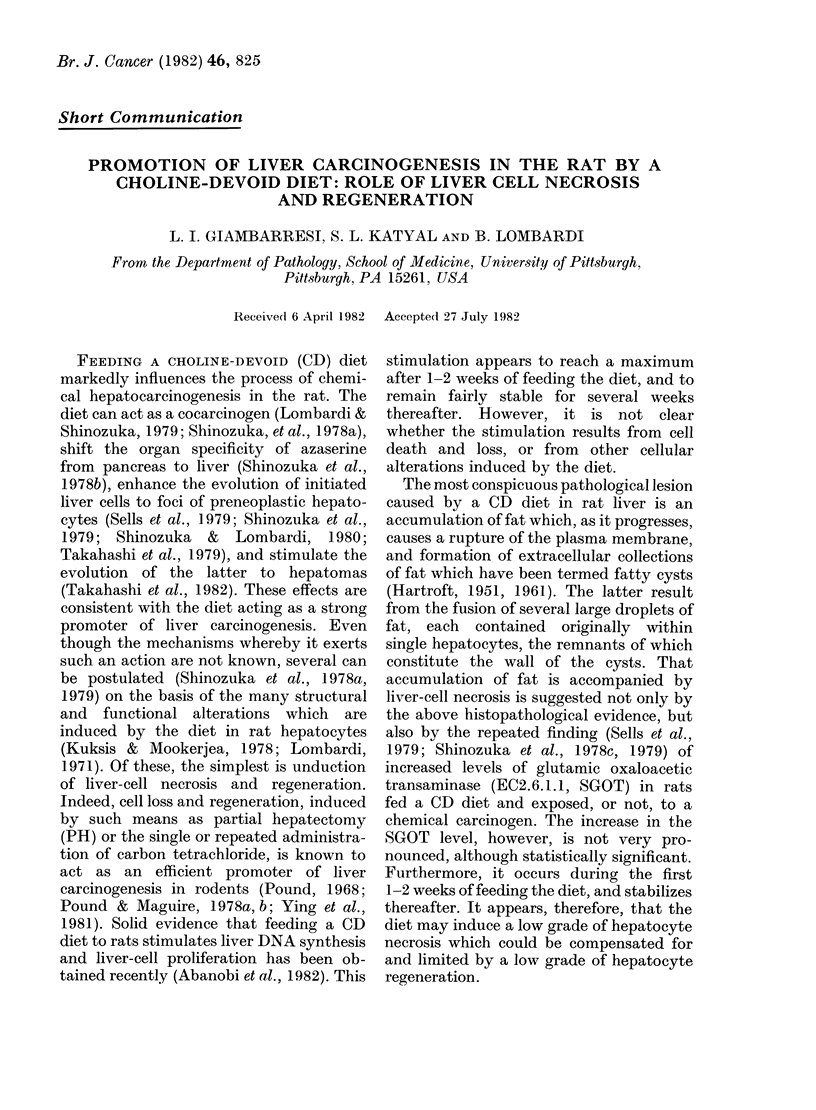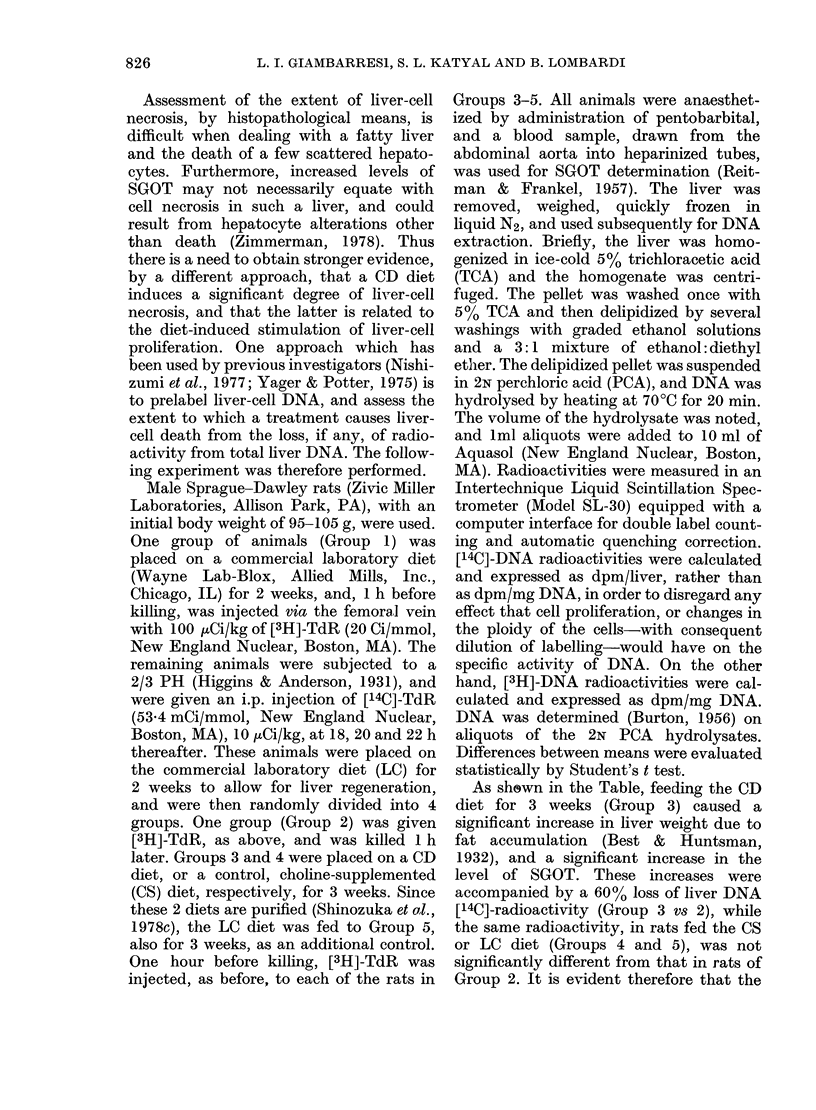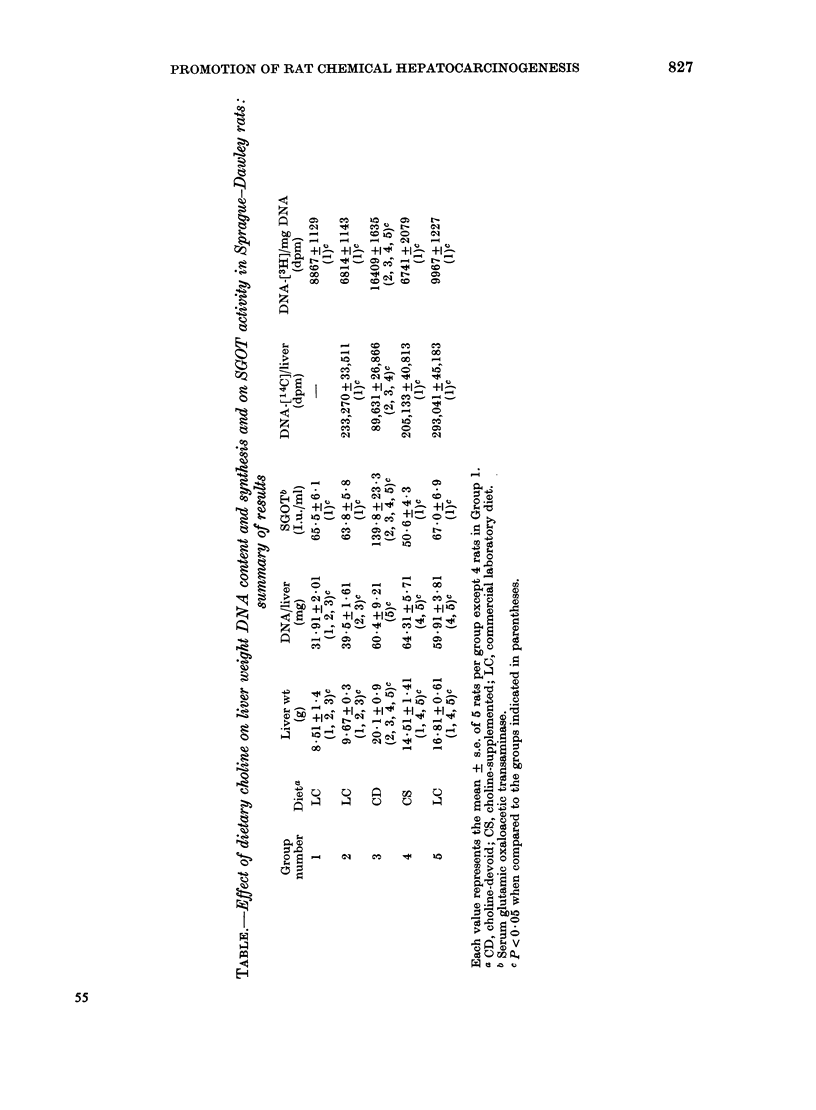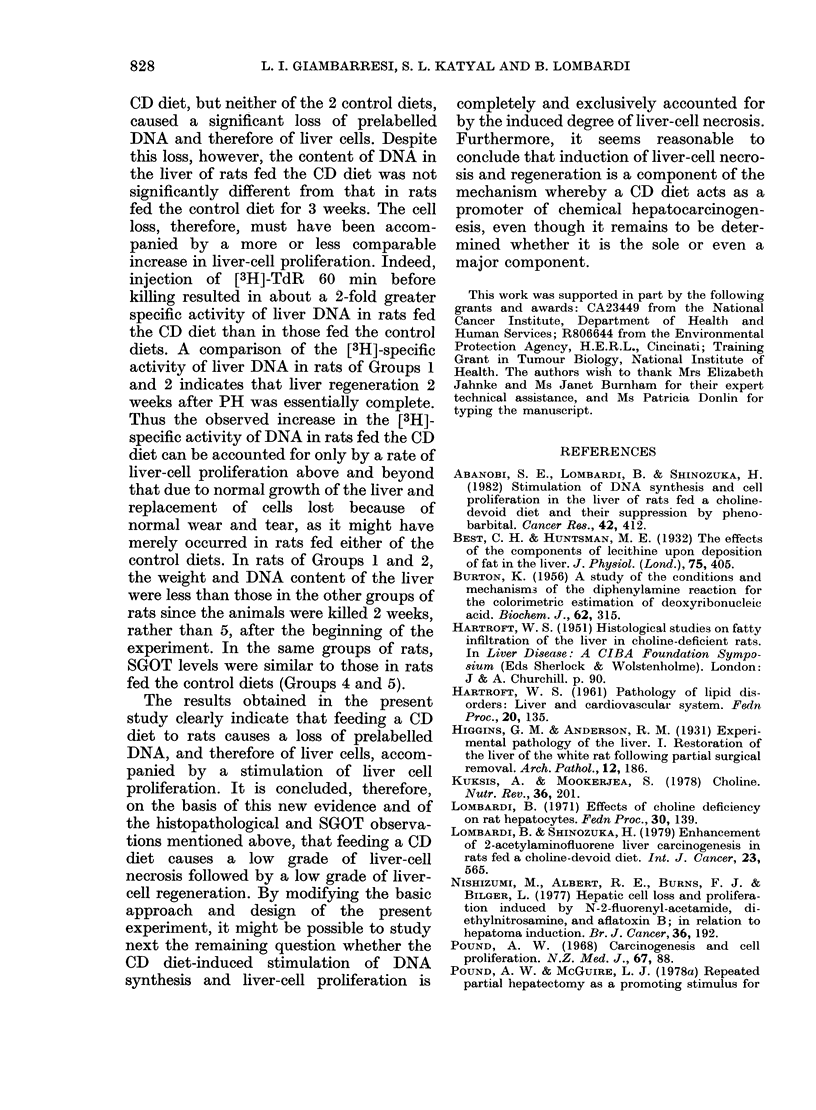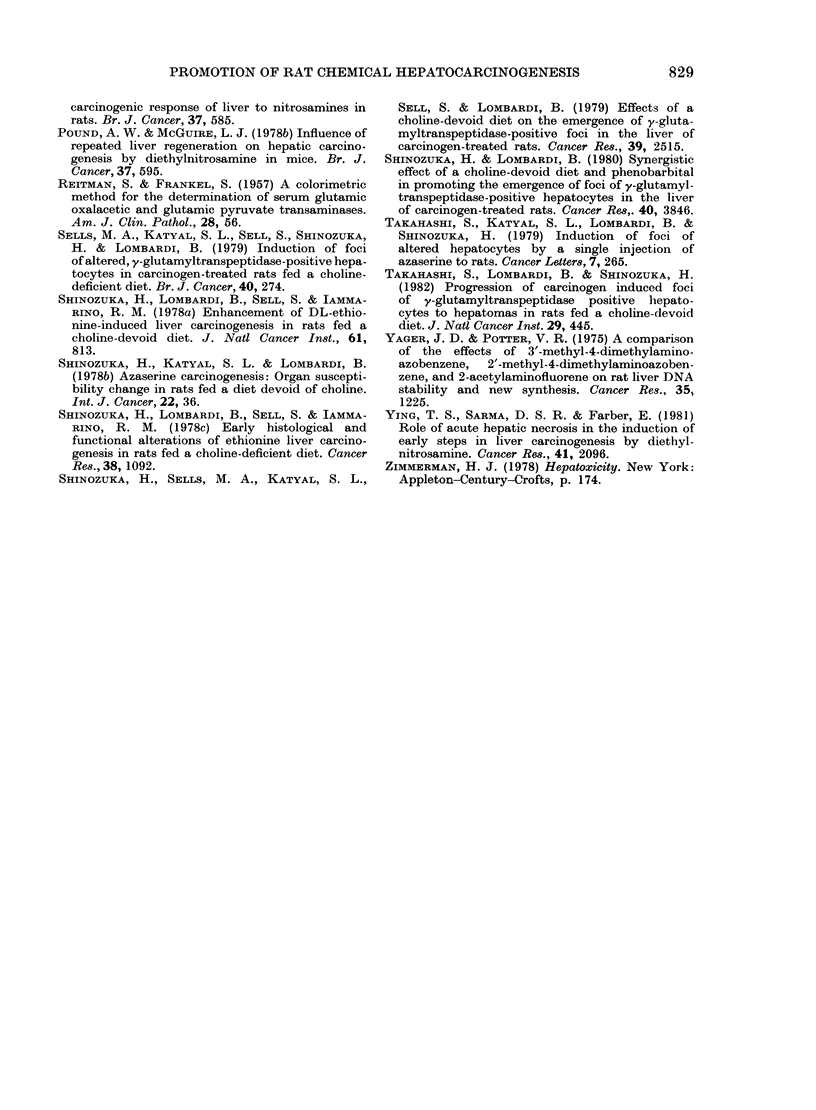# Promotion of liver carcinogenesis in the rat by a choline-devoid diet: role of liver cell necrosis and regeneration.

**DOI:** 10.1038/bjc.1982.278

**Published:** 1982-11

**Authors:** L. I. Giambarresi, S. L. Katyal, B. Lombardi


Br. J. Cancer (1982) 46, 825

*Short Communication*

### PROMOTION OF LIVER CARCINOGENESIS IN THE RAT BY A CHOLINE-DEVOID DIET: ROLE OF LIVER CELL NECROSIS AND REGENERATION

L. I. GIAMBARRESI, S. L. KATYAL AND B. LOMBARDI

*From the Department of Pathology, School of Medicine, University of Pittsburgh, Pittsburgh, PA* 15261, *USA*

Received 6 April 1982 Accepted 27 July 1982

FEEDING A CHOLINE-DEVOID (CD) diet markedly influences the process of chemical hepatocarcinogenesis in the rat. The diet can act as a cocarcinogen (Lombardi & Shinozuka, 1979; Shinozuka, *et al.*, 1978a), shift the organ specificity of azaserine from pancreas to liver (Shinozuka *et al.*, 1978*b*), enhance the evolution of initiated liver cells to foci of preneoplastic hepatocytes (Sells *et al.*, 1979; Shinozuka *et al.*, 1979; Shinozuka & Lombardi, 1980; Takahashi *et al.*, 1979), and stimulate the evolution of the latter to hepatomas (Takahashi *et al.*, 1982). These effects are consistent with the diet acting as a strong promoter of liver carcinogenesis. Even though the mechanisms whereby it exerts such an action are not known, several can be postulated (Shinozuka *et al.*, 1978*a*, 1979) on the basis of the many structural and functional alterations which are induced by the diet in rat hepatocytes (Kuksis & Mookerjea, 1978; Lombardi, 1971). Of these, the simplest is unduction of liver-cell necrosis and regeneration. Indeed, cell loss and regeneration, induced by such means as partial hepatectomy (PH) or the single or repeated administration of carbon tetrachloride, is known to act as an efficient promoter of liver carcinogenesis in rodents (Pound, 1968; Pound & Maguire, 1978*a*, *b*; Ying *et al.*, 1981). Solid evidence that feeding a CD diet to rats stimulates liver DNA synthesis and liver-cell proliferation has been obtained recently (Abanobi *et al.*, 1982). This stimulation appears to reach a maximum after 1–2 weeks of feeding the diet, and to remain fairly stable for several weeks thereafter. However, it is not clear whether the stimulation results from cell death and loss, or from other cellular alterations induced by the diet.

The most conspicuous pathological lesion caused by a CD diet in rat liver is an accumulation of fat which, as it progresses, causes a rupture of the plasma membrane, and formation of extracellular collections of fat which have been termed fatty cysts (Hartroft, 1951, 1961). The latter result from the fusion of several large droplets of fat, each contained originally within single hepatocytes, the remnants of which constitute the wall of the cysts. That accumulation of fat is accompanied by liver-cell necrosis is suggested not only by the above histopathological evidence, but also by the repeated finding (Sells *et al.*, 1979; Shinozuka *et al.*, 1978*c*, 1979) of increased levels of glutamic oxaloacetic transaminase (EC2.6.1.1, SGOT) in rats fed a CD diet and exposed, or not, to a chemical carcinogen. The increase in the SGOT level, however, is not very pronounced, although statistically significant. Furthermore, it occurs during the first 1–2 weeks of feeding the diet, and stabilizes thereafter. It appears, therefore, that the diet may induce a low grade of hepatocyte necrosis which could be compensated for and limited by a low grade of hepatocyte regeneration.

Assessment of the extent of liver-cell necrosis, by histopathological means, is difficult when dealing with a fatty liver and the death of a few scattered hepatocytes. Furthermore, increased levels of SGOT may not necessarily equate with cell necrosis in such a liver, and could result from hepatocyte alterations other than death (Zimmerman, 1978). Thus there is a need to obtain stronger evidence, by a different approach, that a CD diet induces a significant degree of liver-cell necrosis, and that the latter is related to the diet-induced stimulation of liver-cell proliferation. One approach which has been used by previous investigators (Nishizumi *et al.*, 1977; Yager & Potter, 1975) is to prelabel liver-cell DNA, and assess the extent to which a treatment causes liver-cell death from the loss, if any, of radioactivity from total liver DNA. The following experiment was therefore performed.

Male Sprague–Dawley rats (Zivic Miller Laboratories, Allison Park, PA), with an initial body weight of 95–105 g, were used. One group of animals (Group 1) was placed on a commercial laboratory diet (Wayne Lab-Blox, Allied Mills, Inc., Chicago, IL) for 2 weeks, and, 1 h before killing, was injected *via* the femoral vein with 100 $\mu$Ci/kg of [$^{3}$H]-TdR (20 Ci/mmol, New England Nuclear, Boston, MA). The remaining animals were subjected to a 2/3 PH (Higgins & Anderson, 1931), and were given an i.p. injection of [$^{14}$C]-TdR (53·4 mCi/mmol, New England Nuclear, Boston, MA), 10 $\mu$Ci/kg, at 18, 20 and 22 h thereafter. These animals were placed on the commercial laboratory diet (LC) for 2 weeks to allow for liver regeneration, and were then randomly divided into 4 groups. One group (Group 2) was given [$^{3}$H]-TdR, as above, and was killed 1 h later. Groups 3 and 4 were placed on a CD diet, or a control, choline-supplemented (CS) diet, respectively, for 3 weeks. Since these 2 diets are purified (Shinozuka *et al.*, 1978*c*), the LC diet was fed to Group 5, also for 3 weeks, as an additional control. One hour before killing, [$^{3}$H]-TdR was injected, as before, to each of the rats in Groups 3–5. All animals were anaesthetized by administration of pentobarbital, and a blood sample, drawn from the abdominal aorta into heparinized tubes, was used for SGOT determination (Reitman & Frankel, 1957). The liver was removed, weighed, quickly frozen in liquid $N_2$, and used subsequently for DNA extraction. Briefly, the liver was homogenized in ice-cold 5% trichloracetic acid (TCA) and the homogenate was centrifuged. The pellet was washed once with 5% TCA and then delipidized by several washings with graded ethanol solutions and a 3:1 mixture of ethanol:diethyl ether. The delipidized pellet was suspended in 2N perchloric acid (PCA), and DNA was hydrolysed by heating at 70°C for 20 min. The volume of the hydrolysate was noted, and 1ml aliquots were added to 10 ml of Aquasol (New England Nuclear, Boston, MA). Radioactivities were measured in an Intertechnique Liquid Scintillation Spectrometer (Model SL-30) equipped with a computer interface for double label counting and automatic quenching correction. [$^{14}$C]-DNA radioactivities were calculated and expressed as dpm/liver, rather than as dpm/mg DNA, in order to disregard any effect that cell proliferation, or changes in the ploidy of the cells—with consequent dilution of labelling—would have on the specific activity of DNA. On the other hand, [$^{3}$H]-DNA radioactivities were calculated and expressed as dpm/mg DNA. DNA was determined (Burton, 1956) on aliquots of the 2N PCA hydrolysates. Differences between means were evaluated statistically by Student's *t* test.

As shown in the Table, feeding the CD diet for 3 weeks (Group 3) caused a significant increase in liver weight due to fat accumulation (Best & Huntsman, 1932), and a significant increase in the level of SGOT. These increases were accompanied by a 60% loss of liver DNA [$^{14}$C]-radioactivity (Group 3 *vs* 2), while the same radioactivity, in rats fed the CS or LC diet (Groups 4 and 5), was not significantly different from that in rats of Group 2. It is evident therefore that the

TABLE.—*Effect of dietary choline on liver weight DNA content and synthesis and on SGOT activity in Sprague–Dawley rats: summary of results*

| Group number | Diet[a] | Liver wt (g) | DNA/liver (mg) | SGOT[b] (I.u./ml) | DNA-[$^{14}$C]/liver (dpm) | DNA-[$^{3}$H]/mg DNA (dpm) |
|---|---|---|---|---|---|---|
| 1 | LC | 8·51 ± 1·4 (1, 2, 3)[c] | 31·91 ± 2·01 (1, 2, 3)[c] | 65·5 ± 6·1 (1)[c] | — | 8867 ± 1129 (1)[c] |
| 2 | LC | 9·67 ± 0·3 (1, 2, 3)[c] | 39·5 ± 1·61 (2, 3)[c] | 63·8 ± 5·8 (1)[c] | 233,270 ± 33,511 (1)[c] | 6814 ± 1143 (1)[c] |
| 3 | CD | 20·1 ± 0·9 (2, 3, 4, 5)[c] | 60·4 ± 9·21 (5)[c] | 139·8 ± 23·3 (2, 3, 4, 5)[c] | 89,631 ± 26,866 (2, 3, 4)[c] | 16409 ± 1635 (2, 3, 4, 5)[c] |
| 4 | CS | 14·51 ± 1·41 (1, 4, 5)[c] | 64·31 ± 5·71 (4, 5)[c] | 50·6 ± 4·3 (1)[c] | 205,133 ± 40,813 (1)[c] | 6741 ± 2079 (1)[c] |
| 5 | LC | 16·81 ± 0·61 (1, 4, 5)[c] | 59·91 ± 3·81 (4, 5)[c] | 67·0 ± 6·9 (1)[c] | 293,041 ± 45,183 (1)[c] | 9967 ± 1227 (1)[c] |

Each value represents the mean ± s.e. of 5 rats per group except 4 rats in Group 1.

[a] CD, choline-devoid; CS, choline-supplemented; LC, commercial laboratory diet.

[b] Serum glutamic oxaloacetic transaminase.

[c] $P < 0·05$ when compared to the groups indicated in parentheses.

CD diet, but neither of the 2 control diets, caused a significant loss of prelabelled DNA and therefore of liver cells. Despite this loss, however, the content of DNA in the liver of rats fed the CD diet was not significantly different from that in rats fed the control diet for 3 weeks. The cell loss, therefore, must have been accompanied by a more or less comparable increase in liver-cell proliferation. Indeed, injection of [$^3$H]-TdR 60 min before killing resulted in about a 2-fold greater specific activity of liver DNA in rats fed the CD diet than in those fed the control diets. A comparison of the [$^3$H]-specific activity of liver DNA in rats of Groups 1 and 2 indicates that liver regeneration 2 weeks after PH was essentially complete. Thus the observed increase in the [$^3$H]-specific activity of DNA in rats fed the CD diet can be accounted for only by a rate of liver-cell proliferation above and beyond that due to normal growth of the liver and replacement of cells lost because of normal wear and tear, as it might have merely occurred in rats fed either of the control diets. In rats of Groups 1 and 2, the weight and DNA content of the liver were less than those in the other groups of rats since the animals were killed 2 weeks, rather than 5, after the beginning of the experiment. In the same groups of rats, SGOT levels were similar to those in rats fed the control diets (Groups 4 and 5).

The results obtained in the present study clearly indicate that feeding a CD diet to rats causes a loss of prelabelled DNA, and therefore of liver cells, accompanied by a stimulation of liver cell proliferation. It is concluded, therefore, on the basis of this new evidence and of the histopathological and SGOT observations mentioned above, that feeding a CD diet causes a low grade of liver-cell necrosis followed by a low grade of liver-cell regeneration. By modifying the basic approach and design of the present experiment, it might be possible to study next the remaining question whether the CD diet-induced stimulation of DNA synthesis and liver-cell proliferation is completely and exclusively accounted for by the induced degree of liver-cell necrosis. Furthermore, it seems reasonable to conclude that induction of liver-cell necrosis and regeneration is a component of the mechanism whereby a CD diet acts as a promoter of chemical hepatocarcinogenesis, even though it remains to be determined whether it is the sole or even a major component.

This work was supported in part by the following grants and awards: CA23449 from the National Cancer Institute, Department of Health and Human Services; R806644 from the Environmental Protection Agency, H.E.R.L., Cincinati; Training Grant in Tumour Biology, National Institute of Health. The authors wish to thank Mrs Elizabeth Jahnke and Ms Janet Burnham for their expert technical assistance, and Ms Patricia Donlin for typing the manuscript.

### REFERENCES

ABANOBI, S. E., LOMBARDI, B. & SHINOZUKA, H. (1982) Stimulation of DNA synthesis and cell proliferation in the liver of rats fed a choline-devoid diet and their suppression by phenobarbital. *Cancer Res.*, **42**, 412.

BEST, C. H. & HUNTSMAN, M. E. (1932) The effects of the components of lecithine upon deposition of fat in the liver. *J. Physiol. (Lond.)*, **75**, 405.

BURTON, K. (1956) A study of the conditions and mechanisms of the diphenylamine reaction for the colorimetric estimation of deoxyribonucleic acid. *Biochem. J.*, **62**, 315.

HARTROFT, W. S. (1951) Histological studies on fatty infiltration of the liver in choline-deficient rats. In *Liver Disease: A CIBA Foundation Symposium* (Eds Sherlock & Wolstenholme). London: J & A. Churchill. p. 90.

HARTROFT, W. S. (1961) Pathology of lipid disorders: Liver and cardiovascular system. *Fedn Proc.*, **20**, 135.

HIGGINS, G. M. & ANDERSON, R. M. (1931) Experimental pathology of the liver. I. Restoration of the liver of the white rat following partial surgical removal. *Arch. Pathol.*, **12**, 186.

KUKSIS, A. & MOOKERJEA, S. (1978) Choline. *Nutr. Rev.*, **36**, 201.

LOMBARDI, B. (1971) Effects of choline deficiency on rat hepatocytes. *Fedn Proc.*, **30**, 139.

LOMBARDI, B. & SHINOZUKA, H. (1979) Enhancement of 2-acetylaminofluorene liver carcinogenesis in rats fed a choline-devoid diet. *Int. J. Cancer*, **23**, 565.

NISHIZUMI, M., ALBERT, R. E., BURNS, F. J. & BILGER, L. (1977) Hepatic cell loss and proliferation induced by N-2-fluorenyl-acetamide, diethylnitrosamine, and aflatoxin B; in relation to hepatoma induction. *Br. J. Cancer*, **36**, 192.

POUND, A. W. (1968) Carcinogenesis and cell proliferation. *N.Z. Med. J.*, **67**, 88.

POUND, A. W. & MCGUIRE, L. J. (1978*a*) Repeated partial hepatectomy as a promoting stimulus for carcinogenic response of liver to nitrosamines in rats. *Br. J. Cancer*, **37**, 585.

POUND, A. W. & MCGUIRE, L. J. (1978*b*) Influence of repeated liver regeneration on hepatic carcinogenesis by diethylnitrosamine in mice. *Br. J. Cancer*, **37**, 595.

REITMAN, S. & FRANKEL, S. (1957) A colorimetric method for the determination of serum glutamic oxalacetic and glutamic pyruvate transaminases. *Am. J. Clin. Pathol.*, **28**, 56.

SELLS, M. A., KATYAL, S. L., SELL, S., SHINOZUKA, H. & LOMBARDI, B. (1979) Induction of foci of altered, γ-glutamyltranspeptidase-positive hepatocytes in carcinogen-treated rats fed a choline-deficient diet. *Br. J. Cancer*, **40**, 274.

SHINOZUKA, H., LOMBARDI, B., SELL, S. & IAMMARINO, R. M. (1978*a*) Enhancement of DL-ethionine-induced liver carcinogenesis in rats fed a choline-devoid diet. *J. Natl Cancer Inst.*, **61**, 813.

SHINOZUKA, H., KATYAL, S. L. & LOMBARDI, B. (1978*b*) Azaserine carcinogenesis: Organ susceptibility change in rats fed a diet devoid of choline. *Int. J. Cancer*, **22**, 36.

SHINOZUKA, H., LOMBARDI, B., SELL, S. & IAMMARINO, R. M. (1978*c*) Early histological and functional alterations of ethionine liver carcinogenesis in rats fed a choline-deficient diet. *Cancer Res.*, **38**, 1092.

SHINOZUKA, H., SELLS, M. A., KATYAL, S. L., SELL, S. & LOMBARDI, B. (1979) Effects of a choline-devoid diet on the emergence of γ-glutamyltranspeptidase-positive foci in the liver of carcinogen-treated rats. *Cancer Res.*, **39**, 2515.

SHINOZUKA, H. & LOMBARDI, B. (1980) Synergistic effect of a choline-devoid diet and phenobarbital in promoting the emergence of foci of γ-glutamyltranspeptidase-positive hepatocytes in the liver of carcinogen-treated rats. *Cancer Res*,. **40**, 3846.

TAKAHASHI, S., KATYAL, S. L., LOMBARDI, B. & SHINOZUKA, H. (1979) Induction of foci of altered hepatocytes by a single injection of azaserine to rats. *Cancer Letters*, **7**, 265.

TAKAHASHI, S., LOMBARDI, B. & SHINOZUKA, H. (1982) Progression of carcinogen induced foci of γ-glutamyltranspeptidase positive hepatocytes to hepatomas in rats fed a choline-devoid diet. *J. Natl Cancer Inst.* **29**, 445.

YAGER, J. D. & POTTER, V. R. (1975) A comparison of the effects of 3′-methyl-4-dimethylaminoazobenzene, 2′-methyl-4-dimethylaminoazobenzene, and 2-acetylaminofluorene on rat liver DNA stability and new synthesis. *Cancer Res.*, **35**, 1225.

YING, T. S., SARMA, D. S. R. & Farber, E. (1981) Role of acute hepatic necrosis in the induction of early steps in liver carcinogenesis by diethylnitrosamine. *Cancer Res.*, **41**, 2096.

ZIMMERMAN, H. J. (1978) *Hepatoxicity*. New York: Appleton–Century–Crofts, p. 174.